# Biological Response Following the Systemic Injection of PEG–PAMAM–Rhodamine Conjugates in Zebrafish

**DOI:** 10.3390/pharmaceutics16050608

**Published:** 2024-04-30

**Authors:** Beatriz Custódio, Patrícia Carneiro, Joana Marques, Victoria Leiro, Ana M. Valentim, Mafalda Sousa, Sofia D. Santos, José Bessa, Ana P. Pêgo

**Affiliations:** 1 i3S—Instituto de Investigação e Inovação em Saúde, Universidade do Porto, R. Alfredo Allen 208, 4200-135 Porto, Portugalsasantos@ineb.up.pt (S.D.S.);; 2 INEB—Instituto Nacional de Engenharia Biomédica, Universidade do Porto, R. Alfredo Allen 208, 4200-135 Porto, Portugal; 3 ICBAS—Instituto de Ciências Biomédicas Abel Salazar, Universidade do Porto, R. Jorge de Viterbo Ferreira 228, 4050-313 Porto, Portugal; 4 IBMC—Instituto de Biologia Molecular e Celular, Universidade do Porto, R. Alfredo Allen 208, 4200-135 Porto, Portugal

**Keywords:** zebrafish, biocompatibility, neurotoxicity, dose response, systemic injection, nanobiomaterials

## Abstract

Numerous therapeutic and diagnostic approaches used within a clinical setting depend on the administration of compounds via systemic delivery. Biomaterials at the nanometer scale, as dendrimers, act as delivery systems by improving cargo bioavailability, circulation time, and the targeting of specific tissues. Although evaluating the efficacy of pharmacological agents based on nanobiomaterials is crucial, conducting toxicological assessments of biomaterials is essential for advancing clinical translation. Here, a zebrafish larvae model was explored to assess the biocompatibility of poly(amido amine) (PAMAM), one of the most exploited dendrimers for drug delivery. We report the impact of a systemic injection of polyethylene glycol (PEG)-modified G4 PAMAM conjugated with rhodamine (Rho) as a mimetic drug (PEG–PAMAM–Rho) on survival, animal development, inflammation, and neurotoxicity. A concentration- and time-dependent effect was observed on mortality, developmental morphology, and innate immune system activation (macrophages). Significant effects in toxicological indicators were reported in the highest tested concentration (50 mg/mL PEG–PAMAM–Rho) as early as 48 h post-injection. Additionally, a lower concentration of PEG–PAMAM–Rho (5 mg/mL) was found to be safe and subsequently tested for neurotoxicity through behavioral assays. In accordance, no significative signs of toxicity were detected. In conclusion, the dose response of the animal was assessed, and the safe dosage for future use in theragnostics was defined. Additionally, new methodologies were established that can be adapted to further studies in toxicology using other nanosystems for systemic delivery.

## 1. Introduction

Systemic injection is the preferred route of administration for many therapeutic and diagnostic compounds, such as drugs and contrast agents, in clinical settings [1]. Due to the widespread and essential use of this administration route, it is imperative to study the impact of different formulations when exploring it.

In the last decades, clinical approaches using nanobiomaterials have been largely investigated in the context of theragnosis. These biomaterials have been studied as carriers of bioactive compounds/molecules due to their capability to tackle problems related to molecule bioavailability, circulation time, passage through barriers, and tissue targeting [2,3]. Besides evaluating the biological performance of nanobiomaterial-based theragnostic formulations, it is essential to perform a toxicological assessment for further clinical translation. Toxicological testing allows not only for a faster translation to clinics but also for the setting of safe dose intervals. Many toxicological studies have been based on in vitro assays [4] that lack the complexity of biological interactions, which can only be mimicked using animal models [5]. Importantly, the prediction of in vivo performance has been pointed out as a bottleneck for clinical translation [6]. Among animal models, the zebrafish has stood out as a non-mammal animal model widely used in ecotoxicology studies [7]. In the context of chemical testing, the Organisation for Economic Cooperation and Development (OECD)’s guidelines include a fish embryo toxicity (FET) test, which consists of exposing zebrafish embryos to chemicals in the swimming media [8,9] and which can be paralleled to toxicity tests in mammals such as mice [10]. Additionally, the zebrafish’s high genetic homology to humans (around 70%) and rapid development of major organs have contributed to its increased popularity in biomedical research [7,11]. For instance, Kadioglu et al. used zebrafish to predict drug toxicity in humans and validate in silico and in vitro results [12]. Moreover, Rizzo et al. showed that in vivo testing using zebrafish embryos not only corroborated in vitro data, but also provided additional information on toxic effects at the level of the whole organism that could not be observed in more simplified in vitro setups [13]. Besides its well-established use in toxicological tests, external development and currently available genetic manipulation tools have contributed to its use for the analysis of pharmacological response over time [14]. It is believed that the establishment of protocols using non-mammal animal models such as zebrafish will help in profiling pharmacological substances and closing the gap between in vitro and in vivo mammalian models, facilitating rapid and effective pre-clinical development [5,9]. However, standard procedures using zebrafish as an animal model typically expose them to compounds through media, presenting challenges in accurately quantifying the amount of nanobiomaterials to which the animals are exposed [15]. To draw translational conclusions on the dose response to tested formulations, it is better to use routes with more control over the material uptake. Additionally, when compared to systemic injection (nanoliter order), exposure through media requires the use of higher amounts of formulations, which can be a drawback when synthesizing and fine-tuning costly compounds.

We have been exploring dendrimers as nanobiomaterials to transport therapeutic compounds to the central nervous system (CNS) [16,17,18]. Dendrimers stand out for their low polydispersity, exhibiting a well-defined and controlled architecture [19], which is constituted by a core and concentric layers of repeating units that define their generation (G). Dendrimers enable the transport of therapeutic and diagnosis compounds, either through the direct modification of their functional groups with their cargo or by the encapsulation of therapeutic compounds within their structure [20]. Here, we use poly(amido amine) (PAMAM) dendrimers as they are one of the most explored due to their commercial availability and low cost [21,22]. Non-modified PAMAM, especially the cationic variant (protonated amine-terminated), has been reported to induce undesired side effects in biological systems [23,24]. In zebrafish, blood clot formation by the creation of fibrin aggregates after systemic injection has been described [25]. But some modifications to PAMAM have already been explored to enable more efficient and safe drug delivery. For instance, modification by the conjugation of poly(ethylene glycol) (PEG) has been widely described to decrease cytotoxicity and increase blood stability and tissue bioavailability, as we previously proved in mice [16].

In this work, we assessed the toxicological effect in zebrafish larvae as a function of the concentration after the pericardial administration of PAMAM dendrimers, functionalized with PEG and conjugated to the fluorophore rhodamine (Rho), which here was used as a mimetic drug (PEG–PAMAM–Rho). Safe dose intervals were defined based on mortality, morphological alterations, inflammation, and behavioral response. Automated quantification protocols were established and explored for an accurate analysis of the toxicological response.

## 2. Materials and Methods

### 2.1. Synthesis of PEG–PAMAM–Rho

PAMAM–NH_2_ G4 dendrimers supplied in a 10% methanol solution (Dendritech, Inc., Midland, MI, USA) were fluorescently labelled with Rho (5(6)-carboxy-X-rhodamine N-succinimidyl ester (sc-210422, Santa Cruz Biotechnology, Dallas, TX, USA). Amine groups from PAMAM–NH_2_ react with the N-succinimidyl ester group from rhodamine, yielding stable amide bonds. Before reaction, the solution of G4 PAMAM–NH_2_ in methanol was concentrated under reduced pressure and then dried under vacuum to remove the methanol to render PAMAM–NH_2_ as a pale yellow oil. After that, the reaction was carried out as follows: 5(6)-carboxy-X-rhodamine N-succinimidyl ester (2.4 mg, 0.0038 mmol) was added dropwise to a PAMAM–NH_2_ solution (36.0 mg, 0.0025 mmol), both in dry dimethyl sulfoxide (DMSO, 1.7 mL final volume) (276855, Sigma-Aldrich, Merck, St. Louis, MO, USA), and the reaction mixture was magnetically stirred for 12 h at room temperature (RT), in the dark, and under an inert argon atmosphere. The resulting mixture was transferred into a dialysis membrane (3500 MWCO, Spectrum Lab, San Francisco, CA, USA) and dialyzed against Type II water for 48 h (changing the water every 3 h, except at the night) until free Rho could not be detected in the eluate. Finally, the resulting solution was freeze-dried yielding the PAMAM–Rho conjugates as a dark-pink powder (34.3 mg, 92% yield), which were stored at −20 °C until further use. The amount of Rho in the conjugate was quantified by ^1^H-NMR (methanol-d4, 400 MHz, Bruker Avance III, Bruker, Billerica, MA, USA). Chemical shifts are reported in ppm (δ units) and were referenced to the residual solvent signals (methanol-d4).

Amine groups from PAMAM–Rho were functionalized with the succinimidyl carboxymethyl ester from mPEG (mPEG-SCM, M_W_ = 2049, Jenkem Technology Co. Ltd., Plano, TX, USA) through stable amide linkages. G4 PAMAM–Rho was reacted with mPEG-SCM to obtain the desired functionalization of PAMAM surface with PEG chains. Briefly, mPEG-SCM (104.9 mg, 0.0512 mmol) was added dropwise to a PAMAM–Rho solution (34.3 mg, 0.0023 mmol), both in dry DMSO (1.5 mL final volume), and the reaction was magnetically stirred overnight (ON) at RT, protected from light, and under an inert argon atmosphere. The resulting mixture was transferred into a dialysis membrane (10,000 MWCO, Spectrum Lab) and purified against Type II water for 72 h (changing the water every 3 h, except at the night) to remove the unreacted mPEG. Finally, the resulting solution was freeze-dried leading to the PEG–PAMAM–Rho conjugates as a dark-pink foaming solid (113.0 mg, 94% yield), which were stored at −20 °C until further use. The PEGylation degree was determined by ^1^H-NMR (D_2_O, 400 MHz). Chemical shifts are reported in ppm (δ units) and were referenced to the HOD signal (D_2_O). 

### 2.2. Characterization of PEG–PAMAM–Rho

The size distribution and particle surface charge of PEG–PAMAM–Rho were measured by dynamic light scattering (DLS) and laser Doppler electrophoresis, respectively, using a Zetasizer Nano ZS (Malvern Instruments, Malvern, Worcestershire, UK). To visualize, measure, count, and characterize PEG–PAMAM–Rho for nanoparticle tracking analysis (NTA), a NanoSight NS300 (Malvern instruments, Malvern, Worcestershire, UK) was used. The lyophilized dendrimer, which was stored at −20 °C, was freshly dissolved in sterile saline solution (0.9% *w*/*v* NaCl) at 0.5 mg/mL and vortexed. DLS measurements were performed at 633 nm with a detection angle of 173°, at room temperature (23 °C), and with 40% humidity. The Smoluchowski model was applied for zeta potential determination, and cumulant analysis was used for mean particle size determination by intensity. The data obtained are the result of three independent experiments with 3 measures each. Data are expressed as mean ± SD. For NTA analysis, the dissolved PEG–PAMAM–Rho suspension was further diluted 1000× in 0.9% NaCl to achieve a particle count within the linear range of 1 × 10^8^ and 1 × 10^9^ particles/mL. Three videos of 30 s were recorded under controlled fluid flow. Camera focus was adjusted to obtain a clear sharp image of the particles (camera level set to 15). The analysis was performed using a 532 nm laser with a 565 nm long-pass filter, the camera level set at 15, and a detection threshold of 3. Each video, with more than 1000 detected tracks, was analyzed using the automatic functions of NTA 3.1 Build 3.1.54 software. Results represent the mean ± SD of three independent analyses.

### 2.3. Zebrafish Models and Husbandry

Different zebrafish lines were used in this study which include wild-type (WT)-AB and a transgenic line with macrophages fluorescently labelled with eGFP (mpeg1:EGFP, kindly provided by Mulero Lab, Department of Cell Biology and Histology Faculty of Biology, University of Murcia, Campus Universitario de Espinardo, Murcia, Spain). Adult zebrafish (*Danio rerio*) were maintained under the following conditions: 28 ± 0.5 °C, pH = 7.3–7.5, 800–900 µs, a 14:10 h light:dark cycle, a recirculating water system, and mechanical and biological filtration (Tecniplast, Buguggiate, Italy). Animals were fed twice a day with a commercial diet (ZEBRAFEED 400–600 μm, Sparos, Olhão, Portugal) and three times a week with artemia (Premium 260 Grade Cysts, Zebrafish Management Ltd., Twyford, UK). All procedures were performed in agreement with the i3S Animal Welfare Bodies and Ethics Committee guidelines, the EU directive (2010/63/EU) and Portuguese law (DL 113/2013). Experiments described here were approved by the Portuguese Veterinary Authorities (DGAV, license reference 0421/000/000/2017).

### 2.4. Embryo and Larvae Collection and Maintenance

Adult zebrafish were crossed at a proportion of 2 males:3 females in a breeding tank for spawning after the room’s light was turned on. Embryos were collected and kept at 28 ± 0.5 °C in E3 medium (5 mM NaCl (sodium chloride, MB15901, Nzytech, Lisbon, Portugal), 0.17 mM KCl (potassium chloride, 2676.298, VWR, Radnor, PA, USA), 0.33 mM CaCl_2_·2H_2_O (calcium chloride, C3881, Sigma-Aldrich), 0.33 mM MgSO_4_·7H_2_O (magnesium sulfate, 63140, Sigma-Aldrich), and 0.01% methylene blue (A1402,0025, Panreac AppliChem, Darmstadt, Germany), at a pH of 7.2) containing 0.01% 1-phenyl-2-thiourea (PTU, #P7629, Sigma-Aldrich). At 72 h post-fertilization (hpf), larvae that did not hatch were dechorionated by mechanical peeling (using tweezers under a stereomicroscope). Larvae after 5 days post-fertilization (dpf) were fed with a commercial diet (ZEBRAFEED 100–200 μm, Sparos).

### 2.5. Nanosystem Administration

At 4 dpf, healthy larvae were injected at the pericardial site with PEG–PAMAM–Rho solutions of 5, 20, and 50 mg/mL (freshly prepared in 0.9% NaCl saline solution). As negative controls, non-injected animals and animals injected with vehicle (0.9% NaCl saline solution) were used. Larvae were anesthetized with 140 mg/L tricaine (MS222; ethyl-3-aminobenzoate methanesulfonate, #E10521, Sigma-Aldrich) [26] in E3 medium with PTU and positioned in custom-design channels molded in 3% (*w*/*v*) agarose in E3 medium (see details in Figure S1, Supplementary Material). Injection was performed by loading injection solutions in borosilicate glass capillaries (GB100F-10P, Science Products GmbH, Hofheim am Taunus, Germany). Capillaries were attached to the micromanipulator (M-152, Narishige, Setagaya, Japan) and injection controlled by a microinjector (IM 300 microinjector, Narishige, Setagaya, Japan). Injection pressure was adjusted to inject 2–5 nL of the tested solutions at the pericardial site. After injection, larvae were transferred to Petri dishes containing fresh E3 with or without PTU medium (for imaging or for behavioral tests, respectively) and incubated at 28 ± 0.5 °C until further analysis.

### 2.6. Survival Quantification

At 4-, 24-, 48-, and 72-h post-injection (hpi), as well as 6 days post-injection (dpi), the number of live animals was quantified (*n* = 128). Median lethal dose (LC50) and dose lethal to 10% of animals (LC10) were calculated by simple logistic regression using GraphPad Prism software (version 10.1.2) as previously described [27], where LC50 and LC10 values correspond to the X value at 50% and 10% of the best-fit values, respectively.

### 2.7. Imaging Acquisition and Processing

Imaging was performed in mpeg1:EGFP larvae maintained in PTU containing E3 medium. At 4, 24, 48, and 72 hpi and 6 dpi larvae were anesthetized with 140 mg/L tricaine and transferred into a 3% agarose-coated Petri dish. Images of the whole animal positioned laterally were acquired under a fluorescence stereomicroscope (Leica M205FA, Leica Microsystems CMS GmbH, Mannheim, Germany) coupled to a digital camera (C11440-42U-USB-102181, Hamamatsu, Shizuoka, Japan). The images were acquired using a 1×/0.06 objective, using empty (brightfield), ET GFP (excitation 470 nm, emission 525 nm, acquire eGFP fluorescence), and ET mCHER (excitation 546 nm, emission 605 nm, acquire rhodamine fluorescence) filter sets. Zoom was adapted to each timepoint for the image to cover the full animal. Images were acquired with image size of 2048 × 2048 pixels using LAS X software (version 2.0.0.14332, Leica Microsystems CMS GmbH).

To convert files acquired in LAS X (.lif) into TIFF files, a Fiji (ImageJ 1.54f) macro (“LIFFtoTIFF_seriesname.ijm” available at https://github.com/nBTTlab/beatrizcustodio_PAMAM, last accessed on 1 April 2024) was used. Following conversion, the Fiji macro was tailored to automatically rotate the animal to a horizontal position and save each acquired channel (brightfield, eGFP, and rhodamine) of the image, segmented animal, and rostral and caudal regions (Fiji macro “ROIRostralCaudal_splitchannels.ijm” available at https://github.com/nBTTlab/beatrizcustodio_PAMAM). Rostral and caudal regions were manually identified based on where the yolk sac starts to elongate (see Figure S2).

#### 2.7.1. Rhodamine Intensity Quantification 

Rhodamine intensity of the whole animal was obtained using a CellProfiler pipeline (Version 4.2.6, Broad Institute Inc., Cambridge, MA, USA). Briefly, brightfield image of the segmented animal was used to identify the animal as an object (“IdentifyPrimaryObjects” function) with a manual threshold of 0.01. Intensity values of rhodamine were obtained from rhodamine channel image by applying “MeasureObjectIntensity” function in the area of the animal object (pipeline “MorphologyAndMpeg_quantification.cppipe” available at https://github.com/nBTTlab/beatrizcustodio_PAMAM).

#### 2.7.2. Developmental Alteration Quantification 

Rotated brightfield images with segmented animal and black background were used to evaluate developmental parameters, such as length, eccentricity, pericardial area, tail-head angle, and yolk sac area. Length and eccentricity were determined using CellProfiler (Version 4.2.6, Broad Institute Inc.). Briefly, the animal was identified as an object (“IdentifyPrimaryObjects” function) with a manual threshold of 0.01, and values were obtained by applying “MeasureObjectSizeShape” function (pipeline “MorphologyAndMpeg_quantification.cppipe” available at https://github.com/nBTTlab/beatrizcustodio_PAMAM). Pericardial area, head–tail angle, and yolk sac area were quantified using Digimizer software (version 4.1.1.0, MedCalc Software, Ostend, Belgium). Measured areas and larvae contour were manually defined.

#### 2.7.3. Macrophage Area Quantification

The images of the animals (mpeg1:EGFP; eGFP channel) were pre-processed with Ilastik software (version 1.3.3post3, European Molecular Biology Laboratory, Heidelberg, Germany) to define probabilities of macrophage fluorescence images. Training was performed by manual segmentation of 8 images of each dataset, defining two categories: macrophages and background. Subsequently, images were renamed with Advanced Renamer software (version 3.87, Hulubulu Software and Kim Jensen) to uniformize them before importing them to CellProfiler (Version 4.2.6, Broad Institute Inc., Cambridge, MA, USA). Whole animal and rostral and caudal regions were identified as objects (“IdentifyPrimaryObjects” function) with a manual threshold of 0.01. Macrophages were identified from obtained Ilastik probabilities images as objects (“IdentifyPrimaryObjects” function) with a manual threshold of 0.25. Area occupied by macrophages was obtained in the whole animal and rostral and caudal regions by applying “MeasureObjectSizeShape” function (pipeline “MorphologyAndMpeg_quantification.cppipe” available at https://github.com/nBTTlab/beatrizcustodio_PAMAM). 

### 2.8. Behavioral Analysis

Behavioral parameters were assessed in WT-AB larvae to evaluate potential alterations on activity/locomotor behavior and anxiety-like behaviors. Both control groups (non-injected and vehicle) and larvae injected with 5 mg/mL PEG–PAMAM–Rho were evaluated at 72 hpi. From the injection day until the behavioral test, larvae were kept in E3 media at 28 ± 0.5 °C with a 14:10 h light:dark cycle, and medium was renewed daily. Animals (*n* = 38) were recorded in an adapted tissue culture in polystyrene 6-well plate under light conditions (details in Figure S3a) for 10 min in a block design experiment. Each block corresponds to a 6-well plate with 2 animals in each treatment. Position of the animals in each plate was randomly assigned using “RAND” function in Excel (Microsoft Office Professional Plus 2016). Six-well plate was placed on top of a light plate and covered by a box which holds a camera (GoPro HERO8) to record the top (Figure S3b,c). This procedure eliminated possible influence of environmental light on video analysis. Following 30 min of larvae habituation, videos of 10 min at 60 frames per second (fps) and 2× zoom were acquired. Behavioral parameter quantification was performed using ANY-maze Video Tracking System (version 7.35, Stoelting Co., Wood Dale, IL, USA). Total distance swum, mean and maximum speed, time spent immobile (sensitivity: 65%, minimum period of immobility: 2000 ms), angular velocity, and distance travelled and time spent in the inner and outer zones of the well were analyzed (for detailed information, see Figure S3d).

### 2.9. Statistical Analysis

All our statistical analyses were performed and our graphs were created using GraphPad Prism software (version 10.1.2). Probability of survival was calculated by Kaplan Meier survival analysis and statistical analysis using log-rank (Mantel–Cox) test. Data normality was verified by Kolmogorov–Smirnov test with and without logarithmic transformation, and a Geisser–Greenhouse correction was used for the homogeneity of variances. When Gaussian distribution was attained, parametric tests were used, namely one- or two-way ANOVA followed by Tukey’s multiple comparison tests. In the absence of Gaussian distribution data fitting, Kruskal–Wallis tests followed by Dunn’s multiple comparisons tests were conducted. A *p*-value below 0.05 was considered statistically significant. 

## 3. Results

### 3.1. PEG–PAMAM–Rho Synthesis and Characterization 

Commercially available G4 PAMAM–NH_2_ was successful functionalized via stable amide bonds with one molecule of Rho and 18 PEG chains (2049 Da) to render the desired conjugate (PEG–PAMAM–Rho), which was characterized by ^1^H-NMR (see Figure S4a,b).

The conjugate was also characterized in terms of its size, polydispersity index (PDI), particle concentration, morphology, and zeta potential. The hydrodynamic size calculated by intensity was found to be 158.0 ± 12.0 nm and by volume was 166.4 ± 5.4 nm, with a PDI of 0.282 ± 0.02 nm (see Figure S4c). The surface charge was positive (+34.5 ± 18.0 mv). To further visualize, measure, count, and characterize PEG–PAMAM–Rho, a NanoSight instrument was used to determine the average size and particle concentration of the conjugate by NTA. This allowed us to assess the abundance of PEG–PAMAM–Rho in the saline solution and to determine the size by a different methodology. In accordance, the conjugate size obtained through this method was found to be 140.3 ± 4.5 nm, which is very close to the values obtained by DLS. As expected, the particles presented a spherical shape (see Video S1), with the concentration of the PEG–PAMAM–Rho suspension found to be 4.16 × 10^11^ ± 0.42 × 10^11^ particles/mL.

### 3.2. Highest Tested PEG–PAMAM–Rho Concentrations Impact the Development and Mortality after Systemic Injection in Zebrafish Larvae

**Figure 1:**
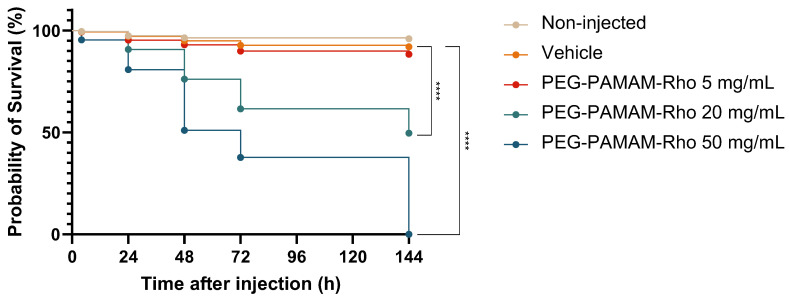
Kaplan Meier survival analysis after PEG–PAMAM–Rho injection. Probability of survival over time depending on the concentration. Animals were analyzed 4 hpi, 24 hpi, 48 hpi, 72 hpi, and 6 dpi (144 hpi). Statistical analysis was performed using log-rank (Mantel–Cox) test. Statistical data presented are the comparison between vehicle control group and other experimental conditions (**** *p* < 0.0001).

We used the rhodamine fluorophore as a cargo/drug mimetic in PEG–PAMAM–Rho, which also allowed us to confirm the success of the injection and track the dendrimers in regards to the time post-administration (see Figure S5). As expected, higher values of rhodamine fluorescence can be detected 4 hpi with the increasing concentration of the injection. Over time, the fluorescence decreased expectedly due to the PEG–PAMAM–Rho distribution and excretion. The increase in injected PEG–PAMAM–Rho concentration was found to compromise the survival of the zebrafish larvae (Figure 1), with no probability of survival at 6 days post-injection (dpi) for the highest injected dose (50 mg/mL). When compared to the injection of the vehicle, only the concentration of 5 mg/mL of PEG–PAMAM–Rho did not significantly impact the survival of the injected larvae. Based on the data represented in Figure 1, the LC50 of pericardially injected PEG–PAMAM–Rho at 6 dpi (144 hpi) was calculated to be 19.16 mg/mL. This value was obtained using a simple logistic regression, a statistical analysis to predict the relationship between the concentration of PEG–PAMAM–Rho injected and the probability of larvae death (see Figure S6). Additionally, we observed that the administration of 5 mg/mL corresponds to an 11.25% probability of death, which is approximately the LC10.

**Figure 2:**
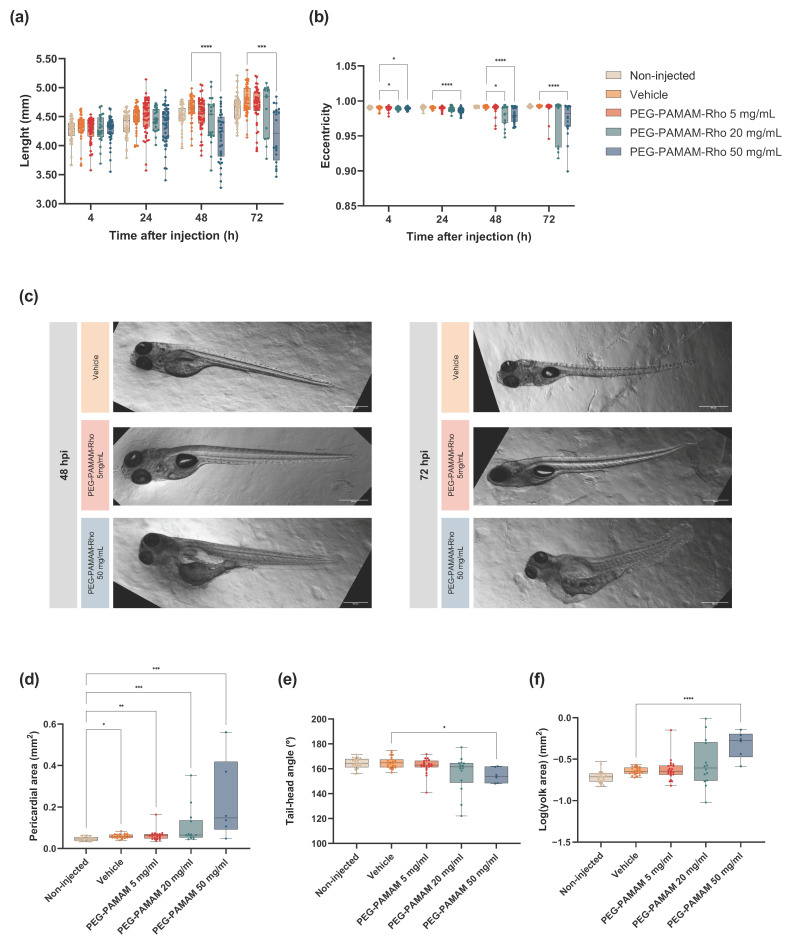
Morphological alterations in development after PEG–PAMAM–Rho systemic injection. Morphological landmarks over time were analyzed including length (**a**) and eccentricity (**b**). At 72 hpi, pericardial area (**d**), tail–head angle (**e**), and yolk area (**f**) were evaluated. Data are presented in box and whisker plots with minimum to maximum shown, and the points represent the data of each individual animal. (**a**,**b**) *n* = 13–48; details in Table S1; (**d**–**f**) *n* = 6–24; details in Table S2. For (**a**–**d**), non-parametric tests were performed using Kruskal–Wallis test followed by Dunn’s multiple comparisons test. For (**f**), one-way ANOVA test was used, followed by Tukey’s multiple comparisons test. Statistical analysis presented is a comparison between the vehicle control group and other experimental conditions in (**a**,**b**,**e**,**f**). In (**d**), statistical analysis presented is a comparison between the non-injected control group and other experimental conditions, as well as the vehicle control group and other experimental conditions (* *p* < 0.05, ** *p* < 0.01, *** *p* < 0.001, **** *p* < 0.0001). (**c**) Representative brightfield images of the malformations observed at 48 hpi and 72 hpi. Scale bar: 500 µm.

In addition to the impact on their survival rate, we observed significant morphological alterations in their development, particularly at the highest concentration of PEG–PAMAM–Rho (50 mg/mL) (Figure 2). To assess this, we measured the length of the segmented animal as well as its eccentricity at various time points (Figure 2a,b). Comparisons were made only within treatments for each observed stage, as morphological alterations are expected over time due to the normal development of zebrafish larvae. Regarding the length of the animal, the larvae injected with 50 mg/mL PEG–PAMAM–Rho were shorter at 48 and 72 hpi compared to those injected with the vehicle. The eccentricity measurement was defined for the segmented animal and quantified by the elongation of an ellipse, with the shape of a circle defined as zero and an ellipse that approximates a line segment defined as one [28]. The results showed that higher concentrations of the PEG–PAMAM–Rho injected solutions altered the morphology of the animals, as they became rounder and less elongated and had lower eccentricity values compared to those injected with the vehicle, which was confirmed by the observation of altered animal shapes (Figure 2c). Statistical differences were particularly observed at all time points between the animals injected with the vehicle and those treated with PEG–PAMAM–Rho 50 mg/mL (Figure 2b).

A further analysis of typical morphological alterations was performed as previously described [29,30,31,32] for six to twenty-four animals per condition at 72 hpi, such as the pericardial area, tail–head angle, and yolk sac area evaluation (Table S2). All groups subjected to injections had a larger pericardial area compared with that of the non-injected animals, showing that this alteration was not induced by the presence of PEG–PAMAM–Rho, but by the injection procedure (Figure 2d). Nevertheless, the impact of the injections is not reflected in other developmental parameters, as the non-injected and vehicle groups had similar features. On the other hand, PEG–PAMAM–Rho at a concentration of 50 mg/mL led to morphological alterations in the tail–head angle and yolk sac area (Figure 2e,f, respectively), consistent with the results observed for length and eccentricity.

### 3.3. A Significant Inflammatory Response Is Observed When Higher PEG–PAMAM–Rho Dosages Are Administered

**Figure 3:**
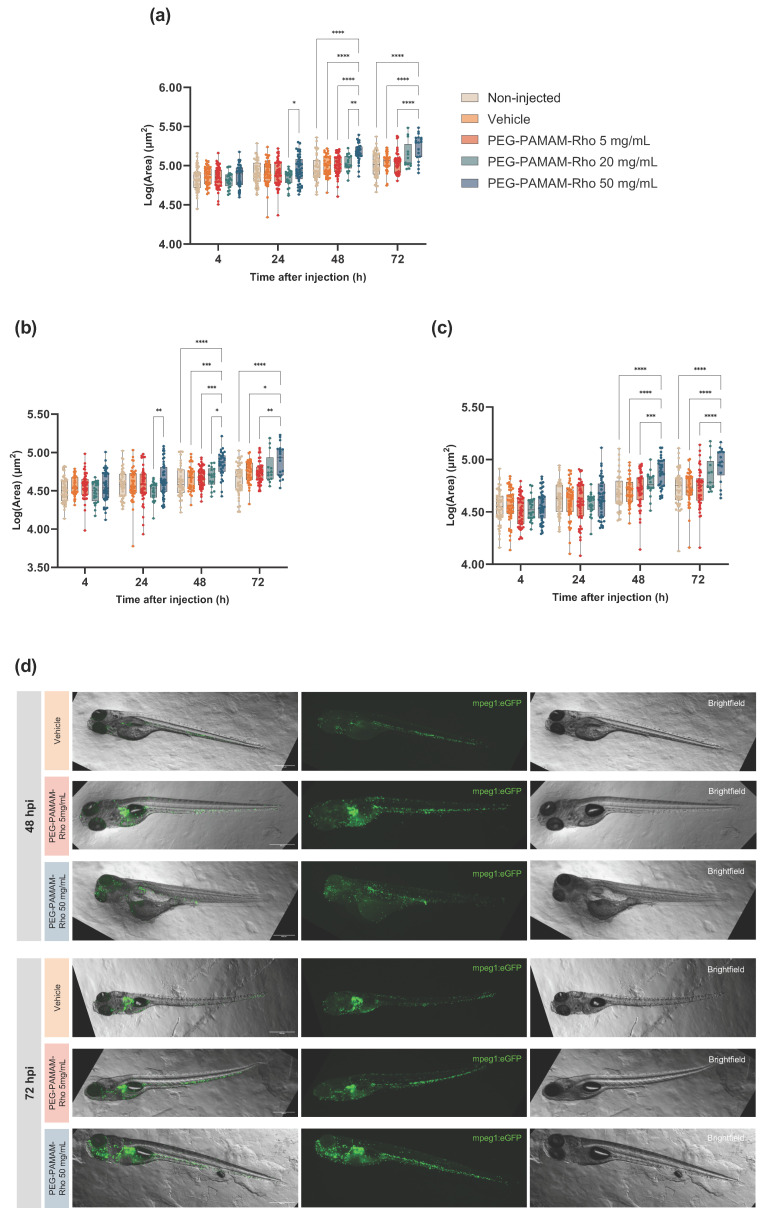
Macrophage response after PEG–PAMAM–Rho injection. Macrophage area in each animal (**a**), rostral area (**b**) and caudal area (**c**). (**d**) Representative images of macrophages (mpeg1:GFP, green) in the larvae (brightfield) at 48 hpi and 72 hpi: left: merged, middle: mpeg1:EGFP (green), right: animal (brightfield). Scale bar: 500 µm. Data are presented in box and whisker plots with minimum to maximum, and the points represent the data of each individual animal (*n* = 13–48; details in Table S1). Data were logarithmically transformed to fit a normal Gaussian distribution. Statistical analysis was performed within timepoints to compare different conditions using two-way ANOVA followed by Tukey’s multiple comparisons test (* *p* < 0.05, ** *p* < 0.01, *** *p* < 0.001, **** *p* < 0.0001).

We evaluated the extent of the inflammatory response by quantifying the macrophages in the animals injected with different PEG–PAMAM–Rho concentrations. As in previous studies [33], we used a macrophage-labeled zebrafish transgenic line (mpeg1:EGFP) which allows for the assessment of the inflammatory response after the nanoformulation injections through the analysis of macrophage production and migration. The quantification was performed using the area occupied by the eGFP fluorescent signal within the animal (Figure 3a) and in different regions of the animal: rostral (Figure 3b) and caudal (Figure 3c). We chose to quantify the presence of macrophages in these two regions (Figure S2) since the injection site is in the pericardial area and the macrophage production occurs in the caudal hematopoietic tissue (CHT). 

The statistical interaction between the time after the injections and the treatment was significant. As shown previously, time differences are expected and verified even within control groups (non-injected and vehicle). This is justified by the animal’s development, which inherently produces macrophages due to immune system establishment and development [34]. As we wanted to study the effect of the factor treatment, we decided to proceed with comparisons between treatments at each timepoint. Here, we report the most relevant differences in the acute response to PEG–PAMAM–Rho formulations. It was observed that there was a significant increase in fluorescence in the whole animal 48 and 72 hpi of the animals injected with PEG–PAMAM–Rho compared to that of animals injected with the vehicle, which might be directly correlated with the number of cells expressing eGFP. This difference was noted in both rostral and caudal regions. 

### 3.4. Behavior Is Not Altered for the Lowest PEG–PAMAM–Rho Formulation Tested

**Figure 4:**
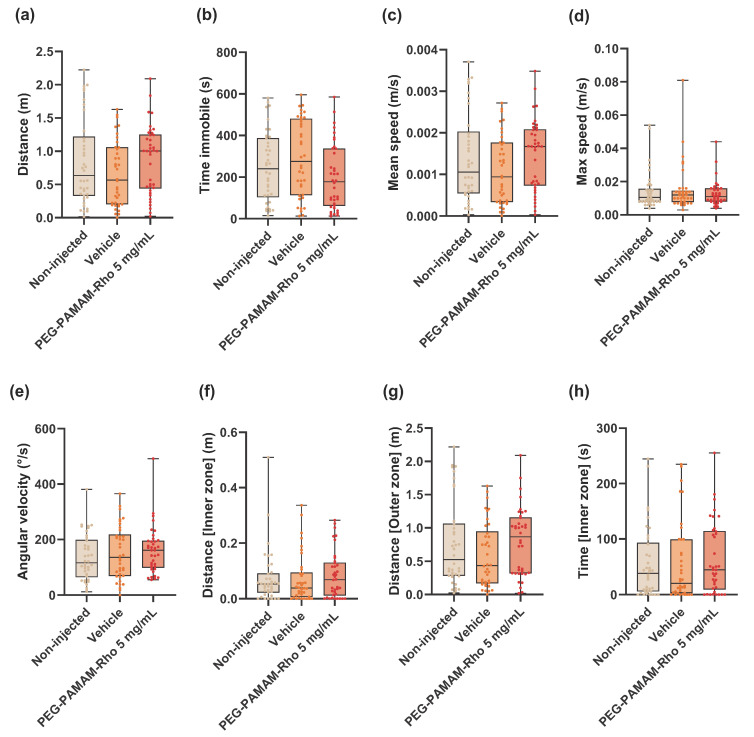
Behavioral alterations 72 hpi after PEG–PAMAM–Rho injection. Parameters related to locomotion, activity (distance in whole well (**a**), time immobile (**b**), mean speed (**c**), maximum speed (**d**), and angular velocity (**e**)), and thigmotaxis (distance travelled in inner (**f**) and outer zones (**g**) and time spent in inner zone (**h**)) were analyzed. Data are presented in box and whisker plots with minimum to maximum, and the points represent the data of each individual animal (*n* = 38). Statistical analysis was performed using Kruskal–Wallis test followed by Dunn’s multiple comparisons test (**a**–**d**,**f**–**h**) or one-way ANOVA test followed Tukey’s multiple comparisons test (**e**). No significant statistical differences were observed between conditions.

Considering the morphological alterations registered after the injection of PEG–PAMAM–Rho at 20 and 50 mg/mL (Figure 2), a behavioral assessment was conducted only in the animals treated with 5 mg/mL PEG–PAMAM–Rho. Morphological alterations have a direct impact on behavioral performance, especially in terms of locomotion/activity. Several behavioral parameters that are usually assessed in toxicological assays in larvae were evaluated [35,36]. The total distance traveled (Figure 4a), time that each animal spent immobile (Figure 4b), mean (Figure 4c) and maximum swimming speed (Figure 4d), and angular velocity (Figure 4e) were similar among all the conditions. To check if the injection or the injected biomaterial interfere with the animals’ levels of anxiety, their occupation of space was also assessed. Two different areas in the well were defined to extrapolate behavioral patterns related to thigmotaxis: an inner zone in the center of the well and an outer zone near the well wall. Thigmotaxis is the preference of an animal to travel and spend more time in a more protected zone, such as near a wall (outer zone: Figure 4g), compared to the inner zone (Figure 4f,h). This behavior is an anxiety-like behavior, and its alteration regarding a control group may indicate alteration on the animal’s anxiety profile. Usually, a more anxious animal spends more time near the wall (outer zone) than control animals [37]. Neither the injection procedure (vehicle) nor injection of PEG–PAMAM–Rho at the concentration of 5 mg/mL induced behavioral alterations regarding locomotion, activity, and anxiety-like behaviors when compared to the control group that was not injected.

## 4. Discussion

With the significant increase in the use of nanomaterials in biomedical applications [38], including for drug delivery, it is important to evaluate the safety of these compounds and their behavior in complex organism systems. In this way, we can improve pre-clinical studies in drug and nanobiomaterials screening and, thus, aid their translation to clinical use [39]. To reach these goals, the establishment, diversification, and refinement of tools and methods in animal models are crucial to improve the reliability of the results and minimize errors in research outcomes.

Zebrafish is a non-mammal animal model widely used in toxicology studies, including the analysis of nanobiomaterials performance, presenting translational results to mammals [6,10,40,41]. Compared to the mouse, one of the most commonly used animal models, zebrafish is a cost-effective model with features that support high-throughput studies [6,11,35]. The external, fast, and well-defined development of zebrafish make their mortality and developmental morphological alterations useful markers for toxicological assessments and enable non-invasive monitoring over time [6,27]. Several studies have reported morphological alterations induced by nanoparticle toxicity [29,30,31,32]. However, most of these reports using zebrafish in early stages of their development exposed the animals to compounds through media [8]. But, using this method of administration, the amount of absorbed compounds is unpredictable and hard to quantify with very few methods established to define this key parameter [15]. This is the case of reported studies for nanosystems based on PAMAM dendrimers in zebrafish, in which the majority follow the media immersion protocol. Here, we assessed the biological response following a pericardial injection of a PEG–PAMAM–Rho conjugate in zebrafish larvae. When a substance is injected into the pericardial cavity of a zebrafish larvae, it can diffuse into the circulatory system and affect the entire organism, including its tissues and organs outside the immediate vicinity of the heart. Thus, while the injection is localized to the pericardial area, the substance can have systemic effects throughout the whole organism.

Toxicological studies in zebrafish have shown that the surface chemistry of nanobiomaterials is one of the major features that impacts their toxicity [42,43]. The cationic nature of amine-terminated PAMAM, under physiological conditions, was described to induce toxic effects as hemolytic and cell toxicity by cell membrane disruption and recognition by the immune system, with PEGylation reducing their toxicity [44]. We have previously shown that the modification of PAMAM with PEG chains has the capacity to reduce toxicity in endothelial and astrocyte cell cultures [16]. Also, PEG modifications are described to increase the circulation time when these materials are injected in vivo [44,45]. Here, the functionalization of G4 PAMAM, through its amine functional groups, with rhodamine and 18 PEG chains (PEGylation in around 28% of its terminal amine groups), resulted in nanosystems with a larger hydrodynamic size when compared to that of the PEG_8_-PAMAM–Rho conjugates we previously reported [16]. This can be justified by the increase in the number of PEGs present at the surface of the dendrimer, which impacts the steric hindrance between PEG chains that become more extended [46]. Nevertheless, one cannot exclude an increased agglomeration of the dendrimers. It should be highlighted, though, that the determined sizes are still in the nanometer range, and the PDI values are below 0.3, which are adequate for in vivo applications [47]. PEGylation is described to shield the cationic nature of the amine-terminated PAMAM [45]. The surface net charge of the prepared nanoformulations was found to be positive, allowing for electrostatic interactions with the negatively charged lipidic cell membranes. In addition, the obtained values for this parameter have been more positive than those previously reported. This can be explained by the higher grafted PEG density, which decreases the non-covalent interactions of the PEG with free PAMAM amines due to the steric hindrance between PEG chains [45]. So, while the charges are shielded by a hydrophilic layer [46], the overall net charge of the nanosystems is still positive. 

Systemically injected compounds are widely used in treatment and diagnosis according to medical guidelines [1]. So, here, we explored the toxicological impact of different concentrations of PEG–PAMAM–Rho after pericardial injections in 4 dpf zebrafish larvae. While the animals are still transparent at this developmental stage, all of their organs are formed. However, the ongoing maturation of the organism still allows for the analysis of the impact of the administered compounds on its development. Also, biological barriers such as the blood–brain barrier (BBB) have already started to mature and have high levels of selectivity [48], similar to those of adults. 

The LC50 of PEG–PAMAM–Rho 6 dpi at the larval stage of the zebrafish was found to be 19.16 mg/mL, which is in contrast to what was reported for the zebrafish exposed to G4 PAMAM in media at the embryonic stage. For the latter, a mortality rate higher than 50% is observed for 0.2 μM (around 0.007 mg/mL) G4 PAMAM when the animals are exposed for 5 days [30]. These differences can be mainly attributed to the shielding effect of PEG, but also to the stage of zebrafish development at the time of exposure or the administration route used, which has been previously described to impact toxicity in zebrafish [49]. The lower rostral–caudal length of larvae injected with 50 mg/mL PEG–PAMAM–Rho can be explained by a toxicological effect that slows the animal’s growth. Also, the less pronounced yolk size decrease in these animals might be related to having a lower food intake and lower energetic consumption. Additionally, the animals’ bent trunk, quantified by the tail–head angle, and the eccentricity decrease after the injection of 50 mg/mL PEG–PAMAM–Rho are possibly correlated with arrested development and muscular degeneration [27]. Previous studies with G4 PAMAM in media have shown time and concentration dependence in inducing the described malformations, as was also observed in our study [30,50,51]. An increase in the pericardial area after the injection (of the vehicle solution or biomaterial) was registered, which can be explained by the mechanical damage or osmotic imbalance provoked by the injection. However, the injection procedure did not impact other developmental parameters.

Zebrafish has emerged as a robust model organism for investigating the innate immune system [52], as key physiological cues and molecular pathways have been evolutionarily conserved in relation to those of mammals [53]. Furthermore, considering that, in its first 4 weeks post-fertilization, a zebrafish has not developed adaptive immunity, the exclusive study of its innate immune system response is possible [34]. This is relevant, as nanobiomaterial/nanoparticle toxicity, biodistribution, and effectiveness are highly dependent on their ability to evade the innate immune system [54,55]. Macrophages are the main players in host defense and highly phagocytic cells [52], and in zebrafish, they are seen in circulation as early as 25 hpf [56]. Thus, the usage of macrophage-labeled zebrafish (mpeg1:EGFP) allowed us to study macrophage production and migration after PEG–PAMAM–Rho injections. These transgenic animals were previously used for a qualitative analysis of macrophage response towards polymeric nanocapsules dependent on the administration route [33]. Here, we explored these modified animals and developed an image analysis pipeline to quantify the macrophage response after PEG–PAMAM–Rho injections. The significant increase in macrophages in the larvae 48 and 72 hpi at the highest concentration (50 mg/mL) might be correlated to a cumulative effect on macrophages. While the rostral region contains the site of injection, at 48 and 72 hpi, the increase in macrophage area is also pronounced in the caudal part, where the macrophage production site (the CHT) is located at this developmental timepoint [57]. The contact with high concentrations of PAMAM may induce the expression of inflammatory cytokines, as previously described for graphene oxide [58], which, in turn, may increase macrophage recruitment and production. Additionally, the positive surface charge of nanobiomaterials is reported to increase macrophage uptake [33]. Nevertheless, lower concentrations of PEG–PAMAM–Rho do not trigger significant macrophage migration and production possibly due to the presence of PEG chains that have previously been shown to decrease the frequency of clotting events, cytotoxicity, and macrophage clearance [16,44,59]. 

Behavioral patterns are dependent on neuromodulator pathways conserved among vertebrates [37]. Previous studies using PAMAM dendrimers report neurotoxic and behavioral effects in a dose-dependent manner after media exposure [29,50,60]. As in a previous study [61], PAMAM dendrimers’ LC10 value was selected in this study to enable further studies. Therefore, a behavioral assessment was performed with the injection of 5 mg/mL PEG–PAMAM–Rho (around LC10) since it did not lead to significant morphological alterations that could compromise the animals’ natural swimming performance at 72 hpi. Locomotor activity, as well as anxiety-like behaviors, were assessed, with no alterations being detected in the analyzed parameters.

It was previously reported that there is some subjectivity in the morphological classification of zebrafish [62]. By establishing protocols to analyze their mortality, morphological alterations, and inflammatory and behavioral response after pericardial injections, it is possible to determine safe formulations and dose intervals. Importantly, the usage in this study of image analysis pipelines that do not mainly rely on user quantification permits the analysis of larger numbers of animals with increased statistical power. In this work, we concluded that lower concentrations of the PEGylated PAMAM are promising carriers for theragnosis molecules, here mimicked by a fluorophore. However, the potential for the surface functionalization of dendrimers offers opportunities for fine-tuning their properties, which must be adjusted to the cargo that is transported, as well as to their final application and target tissue. This includes adjusting the density of PEG grafting or tethering other biocompatible moieties, such as amino acids [63]. These modifications can significantly enhance the biocompatibility and bio-physicochemical characteristics (e.g., size and charge) of these nanosystems, particularly in the development of theragnostic approaches. Furthermore, the explored methodologies provide valuable tools to screen and fine-tune nanoformulations and define safe dose intervals after systemic injections. This will contribute to the early prediction of possible toxicological issues of nanobiomaterials and to an efficient translation to mammals.

## Data Availability

The raw data supporting the conclusions of this article will be made available by the authors, upon request.

## Supplementary Materials

The following supporting information can be downloaded at: https://www.mdpi.com/article/10.3390/pharmaceutics16050608/s1, Figure S1: 3D printed mold representation to create channels for larvae positioning for pericardial injection. The designed mold fits a standard a 75 mm Petri dish; Figure S2: Segmented whole-animal with de definition of rostral and caudal regions used to analyze macrophages distribution; Figure S3: Behavioral setup; Figure S4: PEG-PAMAM-Rho characterization; Figure S5: Rhodamine intensity over time after PEG-PAMAM-Rho injection; Figure S6: Graph representation of the simple logistic regression to calculate median lethal concentration (LC50) at 6 days post-injection; Table S1: Detailed description of individuals (n) per treatment and timepoint that survived and were, subsequently, analyzed for morphology and macrophage area assessment; Table S2: Detailed description of individuals (n) per treatment at 72 hpi that survived and were subsequently analyzed for morphological parameters as pericardial area, tail-head angle, and yolk area; Video S1: Recorded video under controlled fluid flow to visualize, measure, count and characterize PEG-PAMAM-Rho by Nanoparticle Tracking Analysis (NTA) using NanoSight NS300 equipment.
