# Supplementary material for: Biological Response Following the Systemic Injection of PEG–PAMAM–Rhodamine Conjugates in Zebrafish
Source: Pharmaceutics. 2024 Apr 30;16(5):608. doi: 10.3390/pharmaceutics16050608 (PMC11125904; doi:10.3390/pharmaceutics16050608)
Supplement: Supplementary file 1 [file pharmaceutics-16-00608-s001.zip › Supplementary Figures and Tables.pdf]

Supplementary Material

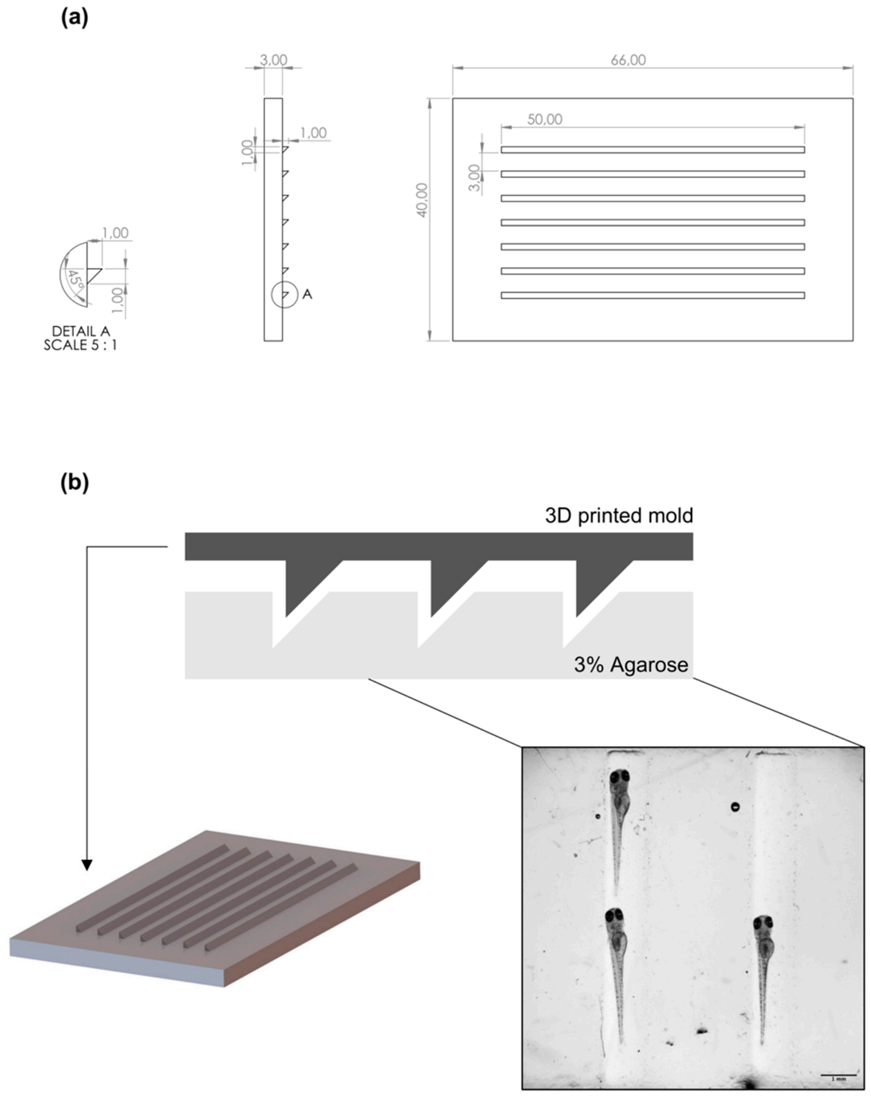

Figure S1. 3D printed mold representation to create channels for larvae positioning for pericardial injection. The designed mold fits a standard a 75 mm Petri dish. **(a)** – Detailed measurements of the design 3D mold in 2D lateral and top representations. File "ZebrafishInjectionMold.STL" available at github.com/nBTTlab/beatrizcustodio_PAMAM. Design and representation done on SOLIDWORKS 2023 (measurements in mm). Printed in a stereolithography Form 3 printer (Formlabs; resolution 25 μm) with Grey V4 resin (Formlabs). **(b)** – Setup representation of the usage of the 3D printed mold to create channels in 3% solidified agarose to position 4 dpf larvae for pericardial injection.

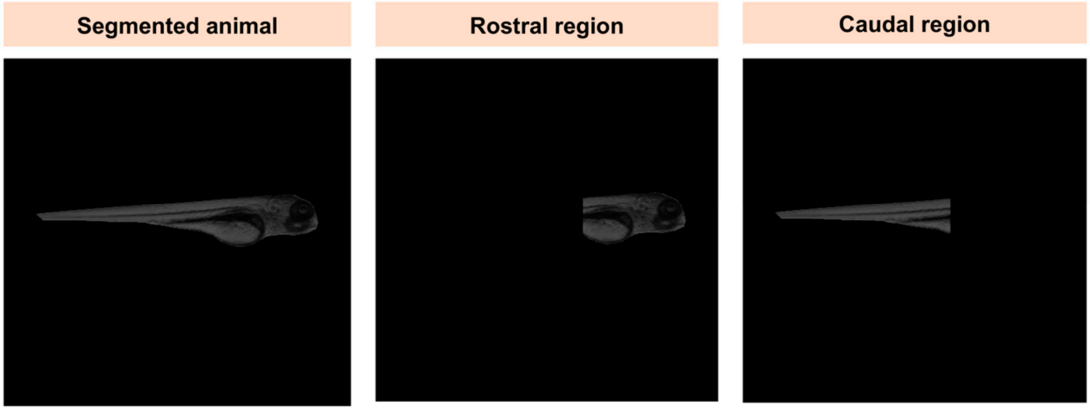

Figure S2. Segmented whole-animal with de definition of rostral and caudal regions used to analyze macrophages distribution.

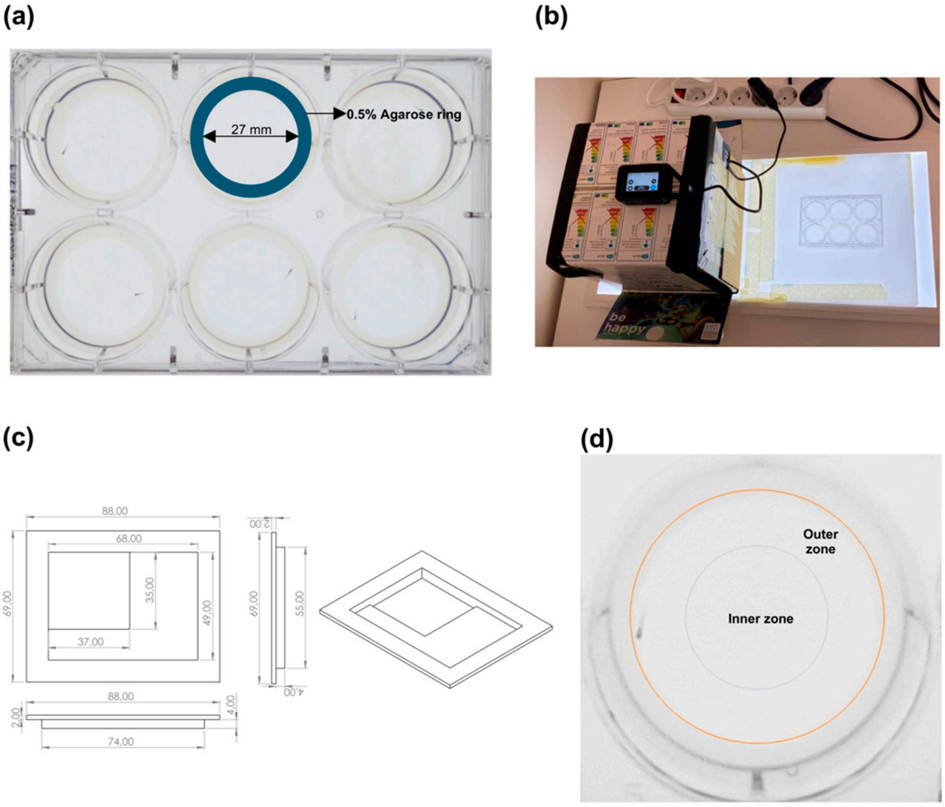

Figure S3. Behavioral setup. **(a)** - Adapted 6-well plate with agarose ring to avoid shadows during image acquisition. A 6-well plate was adapted by adding 5 mL of 0,5% (w/v) agarose in system water. When solidified, a sharp stainless-steel ring (27 mm diameter) was used to create a circular swimming area. Larvae were placed in separate wells with 2 mL of system water. **(b)** – Recording setup of the zebrafish larvae in a 6-well plate. **(c)** – 3D printed GoPro HERO8 adaptor detailed representation; this was printed to attach the camera to the box, allowing a stable recording; File "FitGoPro.STL" available at available at github.com/nBTTlab/beatrizcustodio_PAMAM. Design and representation done on SOLIDWORKS 2023 (measurements in mm) and printed in a stereolithography Form 3 printer (Formlabs; resolution 25 μm) with Grey V4 resin (Formlabs). **(d)** – Defined well areas as inner (16 mm diameter) and outer zones (27 mm diameter) for behavioral analysis.

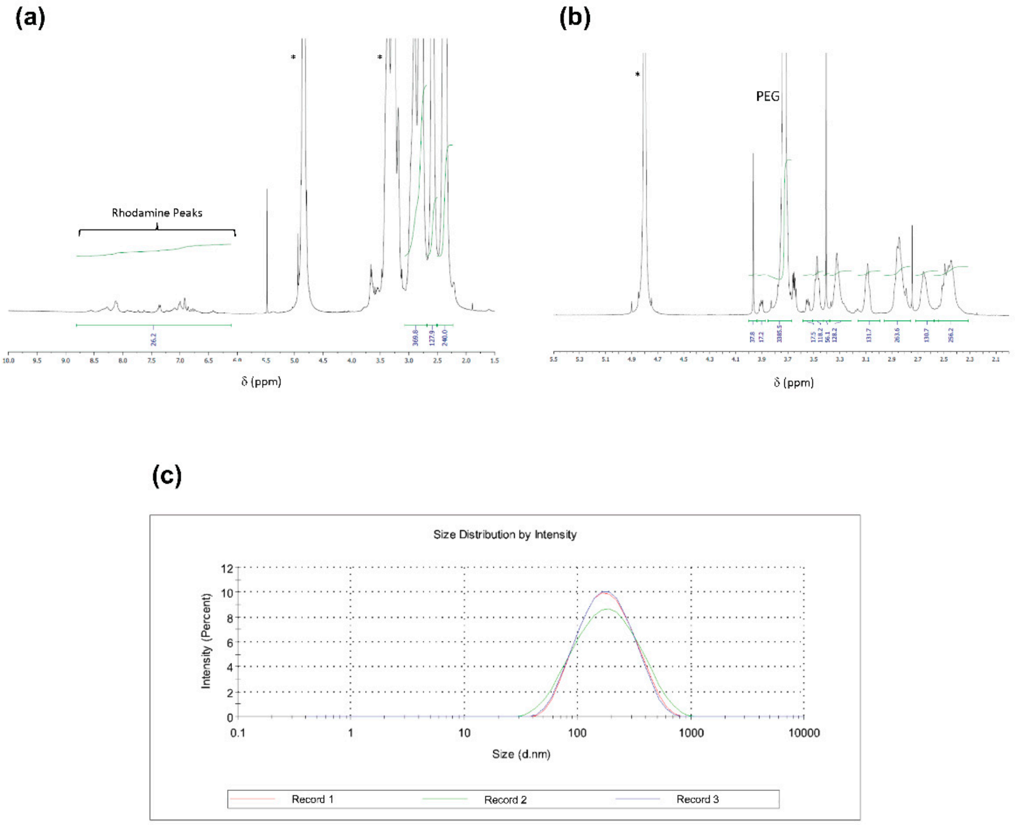

Figure S4. PEG-PAMAM-Rho characterization **(a)** – ^{1}H NMR spectrum (400 MHz, metanol-d4) of PAMAM-NH2 labelled with 5(6)-carboxy-X-rhodamine (PAMAM-Rho). (Solvent peaks labeled as * in the spectrum). **(b)** – ^{1}H NMR spectrum (400 MHz, D_2O) of PEG-PAMAM-Rho. (Solvent peaks labeled as * in the spectrum). Due to the hydrophobicity of rhodamine, its peaks are not clearly detected in D_2O, so this part of the spectrum is not showed here for better visualization of the signals of interest in this compound. **(c)** – Representative graph of size distribution by intensity of the hydrodynamic diameter of PEG-PAMAM-Rho in 0.9% NaCl distribution obtained by DLS.

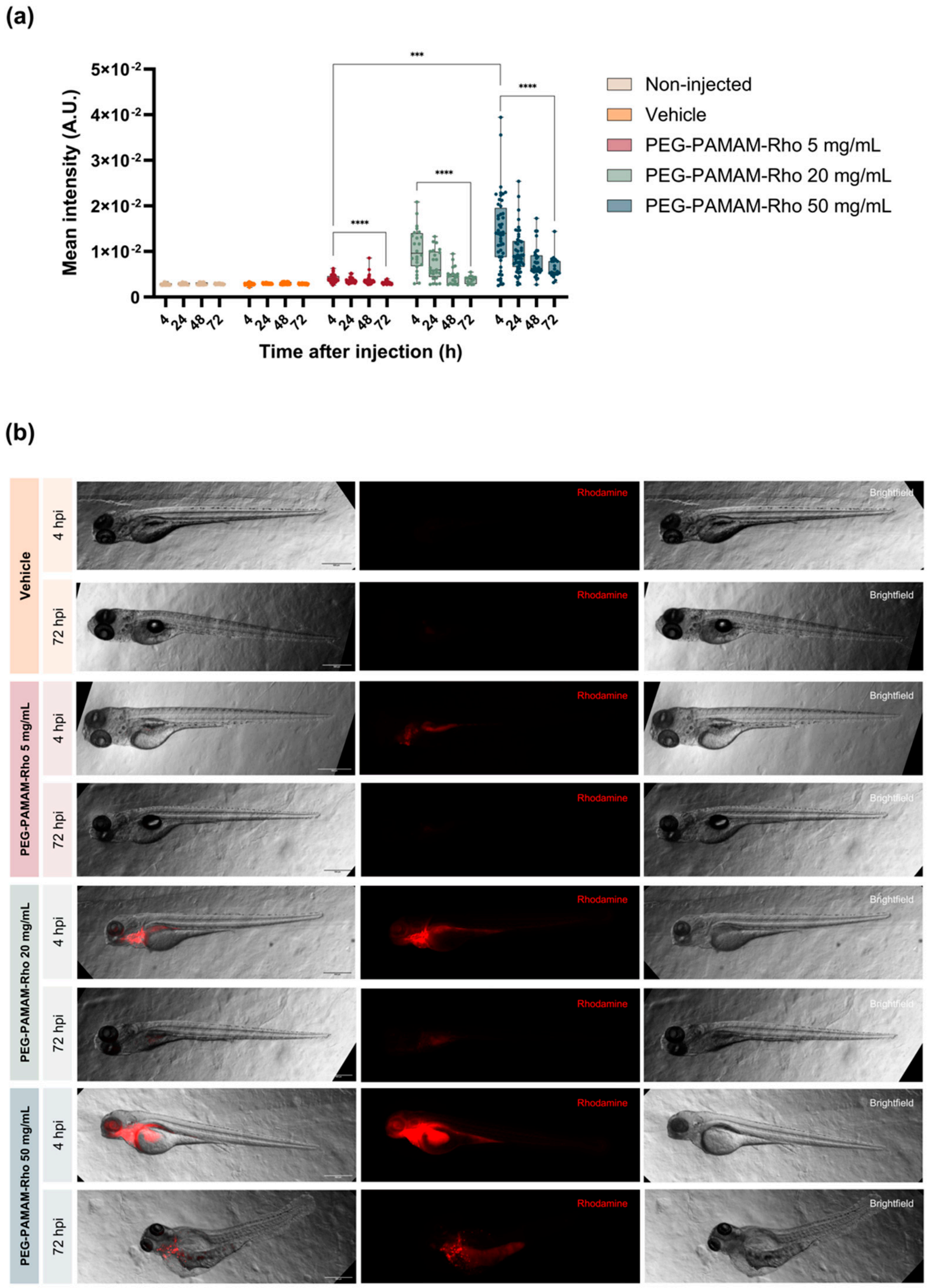

Figure S5. Rhodamine intensity over time after PEG-PAMAM-Rho injection. **(a)** – Fluorescence intensity at different timepoints and conditions. Data is presented in box and whisker plots with minimum to maximum, with the points representing the data of each individual animal (n=13-48, details in Table S1). Statistical analysis was done by Kruskal-Wallis test followed by Dunn's multiple comparisons test to compare 4h vs 72h within each condition and to compare the 4h data of the groups injected with PEG-PAMAM-Rho (***, $p<0.001$ and ****, $p<0.0001$). **(b)** - Representative images of rhodamine (red) linked to PEG-PAMAM-Rho localization in the animal (brightfield) at 4 hpi and 72hpi: left: merged, middle: rhodamine (red), right: animal (brightfield). Scale bar: 500 μm.

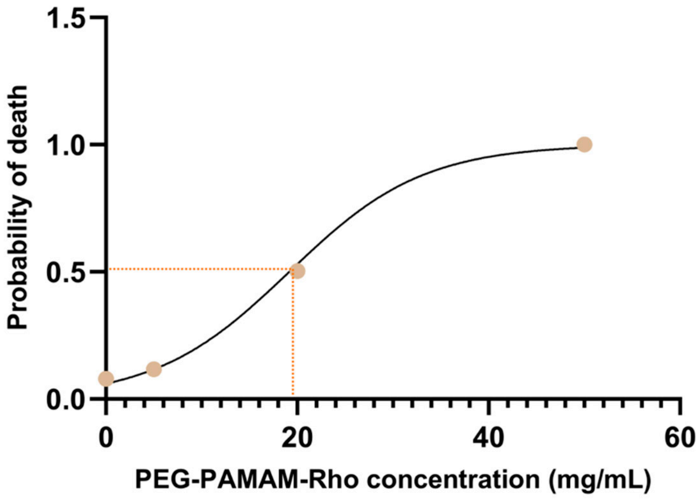

Figure S6. Graph representation of the simple logistic regression to calculate median lethal concentration (LC50) at 6 days post-injection.

Table S1. Detailed description of individuals (n) per treatment and timepoint that survived and were, subsequently analyzed for morphology and macrophage area assessment.

Treatment	4 hpi	24 hpi	48 hpi	72 hpi
Non-injected	47	47	38	47
Vehicle	47	46	37	44
PEG-PAMAM-Rho 5 mg/mL	48	47	48	46
PEG-PAMAM-Rho 20 mg/mL	24	23	18	13
PEG-PAMAM-Rho 50 mg/mL	48	48	33	19

Table S2. Detailed description of individuals (n) per treatment at 72hpi that survived and were subsequently analyzed for morphological parameters as pericardial area, tail-head angle, and yolk area.

Treatment	72 hpi
Non-injected	24
Vehicle	24
PEG-PAMAM-Rho 5 mg/mL	22
PEG-PAMAM-Rho 20 mg/mL	14
PEG-PAMAM-Rho 50 mg/mL	6

Video S1. Recorded video under controlled fluid flow to visualize, measure, count and characterize PEG-PAMAM-Rho by Nanoparticle Tracking Analysis (NTA) using NanoSight NS300 equipment.
